# Maternal anemia across pregnancy in a multi-ethnic tertiary maternity center in a Singaporean population: prevalence, risk factors, and pregnancy outcomes

**DOI:** 10.3389/fmed.2026.1816149

**Published:** 2026-05-07

**Authors:** Hong Min Shaye Peng, Simone Meiqi Ong, Huixin Huang, Hari Yuvaraj, Shau Khng Lim

**Affiliations:** 1 Yong Loo Lin School of Medicine, National University of Singapore, Singapore, Singapore; 2 Department of Obstetrics & Gynaecology, Monash Health, Dandenong Hospital, Dandenong, VIC, Australia; 3 Department of Obstetrics & Gynaecology, Singapore General Hospital, Singapore, Singapore

**Keywords:** anemia in pregnancy, anemia screening, antenatal care, epidemiology, maternal anemia

## Abstract

**Objectives:**

Maternal anemia affects 36 to 50% among pregnant women worldwide. Despite its prevalence and potential harm, contemporary data across different trimesters in multi-ethnic Asian populations remain limited. This retrospective study investigates the prevalence, risk factors and outcomes associated with maternal anemia among 1,005 mothers at a tertiary hospital in Singapore across the first (T1), second (T2), and third (T3) trimesters.

**Methods:**

We conducted a retrospective cohort study of 1,005 pregnant women receiving antenatal care at Singapore General Hospital between 2016 and 2017. Anemia was defined using World Health Organization trimester-specific hemoglobin thresholds. Associations between demographic and clinical factors and maternal anemia were assessed using univariate and multivariable logistic regression adjusted for ethnicity and body mass index (BMI). Maternal and neonatal outcomes were summarized descriptively.

**Results:**

Maternal anemia was identified in 664 women (66.2%) at any point during pregnancy. Prevalence was the highest in T1 (36.9%) and T2 (37.0%), and the lowest in T3 (23.4%). After adjustment, advanced maternal age (≥35 years) was independently associated with T1 anemia (adjusted odds ratio [aOR] 1.40, 95% CI 1.00–1.96). Ethnicity was the strongest predictor across pregnancy, with significantly higher odds among Indian (T1 aOR 3.40; T2 aOR 3.50; T3 aOR 14.1) and Malay women (T1 aOR 2.60; T2 aOR 2.70; T3 aOR 7.80) compared with Chinese women. Maternal and neonatal complications were uncommon overall (2.2 and 0.7%, respectively).

**Conclusion:**

Maternal anemia was highly prevalent in this multi-ethnic tertiary obstetric cohort, particularly in early pregnancy and among Indian and Malay women. These findings support early risk stratification and sustained antenatal surveillance, alongside targeted nutritional counseling and education for higher-risk groups, to reduce preventable maternal and neonatal morbidity.

## Introduction

Anemia, characterized by a hemoglobin (Hb) level below the normal range, reduces the oxygen-carrying capacity of red blood cells (1) and remains one of the most common medical conditions affecting pregnancy. Pregnant women are particularly susceptible due to the physiological demands of gestation (2). According to the WHO, anemia in pregnancy is defined as Hb < 11 g/dL in T1 and T3 and Hb < 10.5 g/dL in T2. Mild, moderate, and severe anemia is also classified as Hb levels 10–10.9 g/dL, 7–9.9 g/dL and <7 g/dL, respectively (3).

Maternal anemia represents a substantial global health challenge, with reported prevalence ranging from 36 to 50% worldwide, affecting both low- and high-income settings (4–6). In Southeast Asia, maternal anemia remains highly prevalent, with reported rates ranging from approximately 25–50% across different populations (4, 7). Maternal anemia has been significantly associated with extremes of maternal age either <20 years (8) or >35 years (9, 10), late booking (defined as first antenatal visit after 12 weeks of gestation) (11, 12), high parity (11, 13), short interpregnancy intervals (14), as well as certain ethnicities such as Indian women (11). Social factors such as low family income, low educational level and unemployment were also identified as factors which contribute to the risk of maternal anemia (11, 15).

Obstetric anemia is defined as anemia occurring during pregnancy. The complications of obstetric anemia impact both mother and child and vary by the trimester of diagnosis and disease severity. Severe obstetric anemia with Hb < 6 g/dL is associated with the adverse effects of poor pregnancy outcome for both mother and child. Maternal morbidity linked to anemia includes systemic issues like high-output cardiac failure, increased risk yet decreased tolerance to hemorrhage, infections, and potentially circulatory shock (16–18). Severe iron deficiency anemia or hemorrhagic anemia may also be presented by obstetric complications, such as placenta previa or abruptio placenta, operative delivery and postpartum hemorrhage. For the fetus, severe obstetric anemia increases the risk of prematurity, spontaneous abortions, low birth weight (LBW), and fetal deaths. In these situations, fetal surveillance and blood transfusion in the mother may be necessary (19, 20).

Despite Singapore’s well-established antenatal care system, contemporary data on maternal anemia remain limited. The most recent local prevalence data were published over three decades ago (21), underscoring the need for updated evidence that reflects current population demographics and clinical practice. Furthermore, trimester-specific patterns of anemia and their associated risk factors have not been well characterized in multi-ethnic Asian populations.

This study aimed to evaluate the prevalence, risk factors, and maternal and neonatal outcomes associated with anemia across all trimesters of pregnancy in a large multi-ethnic cohort at Singapore General Hospital. By identifying high-risk groups and trimester-specific trends, this study seeks to inform targeted antenatal screening and preventive strategies applicable to similar high-income, multi-ethnic settings.

## Materials and methods

### Study design and recruitment

This was a retrospective cohort study conducted at Singapore General Hospital, Singapore’s largest hospital. Between 2016 and 2017, 1,005 pregnant women were included. Eligible participants were Singapore citizens or permanent residents aged 17–50 years, with biparentally homogeneous ethnicity (Chinese, Malay, Indian, or others, e.g., Caucasian). All women conceived naturally without reproductive assistance. Ethical approval was obtained from the SingHealth Centralised Institutional Review Board (CIRB 2019/2844). Informed written consent was also taken from all women prior to recruitment. For participants below 18 years of age, informed consent was obtained from a parent or legal guardian in accordance with institutional ethical guidelines.

### Anemia screening

Anemia screening was performed at the booking visit as part of routine first-trimester antenatal blood investigations. The timing of this visit varied depending on when women first booked antenatal care. For mothers identified with risk factors for anemia (e.g., history of anemia, high parity, or thalassemia trait), follow-up complete blood counts (CBCs) were performed in the second and third trimesters to allow closer surveillance.

### Data collection

Demographic and clinical data were extracted from the Citrix Service Control Manager (SCM) database. Variables included maternal age, ethnicity, body mass index (BMI), thalassaemia carrier status, smoking status, and prior history of anemia. BMI was calculated using weight recorded at the first antenatal (booking) visit, which served as a proxy for pre-pregnancy weight. Obesity was defined as BMI ≥ 30 kg/m^2^ based on World Health Organization criteria for Asian populations.

Obstetric outcomes (e.g., antepartum hemorrhage, preterm labor, postpartum hemorrhage, and placental abnormalities) and neonatal outcomes (e.g., low birth weight, prematurity, neonatal intensive care unit admission, neonatal jaundice, and respiratory distress) were also recorded. Hematological parameters, including hemoglobin (Hb), mean corpuscular hemoglobin concentration (MCHC), and mean corpuscular volume (MCV), were obtained from routine complete blood count testing.

### Statistical analysis

Continuous variables were summarized as mean ± standard deviation (SD) or median (interquartile range, IQR), and categorical variables as frequencies and percentages. Associations between maternal anemia and potential risk factors were examined using Chi-square, Fisher’s exact, independent *t*, or Mann–Whitney *U* tests, as appropriate.

Multivariable logistic regression was used to estimate adjusted odds ratios (aORs) with 95% confidence intervals (CIs) for key predictors, including advanced maternal age (≥35 years), ethnicity, and BMI. Firth bias-reduced logistic regression was applied to address small-sample bias. Variables with sparse data (thalassaemia, smoking, and prior anemia) were excluded from regression analysis.

Maternal and neonatal outcomes were reported descriptively across the entire pregnancy. Due to incomplete hemoglobin data across all trimesters and low event rates, comparative analyses between anemic and non-anemic groups, as well as stratification by anemia severity, were not performed as these would not yield reliable estimates. Statistical significance was defined as two-tailed *p* < 0.05. Analyses were performed using IBM SPSS Statistics Version 29.01.0.

## Results

Characteristics of our maternal anemia patients are depicted in Table 1.

**Table 1:** Characteristics of maternal anemia patients (*N* = 1,005).

Characteristic	*N* (%)
Prevalence of anemia	
T1	371 (36.9%)
T2	372 (37%)
T3	233 (23.4%)
Time of anemia diagnosis	
T1	368 (36.6%)
T2	218 (21.7%)
T3	79 (7.9%)

Characteristic	Median (IQR), Mean ± SD
Haemoglobin – Hb (g/dL)	
T1	12.3 (8.4), 12.2 ± 1.17
T2	11.3 (9.4), 11.1 ± 1.36
T3	12.0 (2.69), 11.9 ± 1.63
Mean corpuscular hemoglobin concentration – MCHC	
T1	33.6 (1.4), 33.4 ± 1.32
T2	33.3 (1.4), 33.1 ± 1.26
T3	33.4 (1.6), 33.2 ± 1.37
Mean corpuscular volume – MCV	
T1	85.7 (7.8), 85.4 ± 24.6
T2	88.2 (9.58), 86.7 ± 7.88
T3	87.7 (9.3), 86.6 ± 21.9

### Prevalence

In our study, the prevalence of maternal anemia in this cohort of 1,005 Singaporean mothers was 66.2% at any point of pregnancy. By trimester, prevalence was highest in T1 (36.9%) and T2 (37.0%), followed by T3 (23.4%). In terms of time of anemia diagnosis, detection rates were the highest in T1 (36.6%) and the lowest in T3 (7.9%).

### Risk factors

Table 2 depicts the prevalence of established risk factors of maternal anemia in our population. Three mothers with a prior history of thalassaemia were excluded from regression analysis. 89.1% of mothers were of Asian descent, with Chinese being the most prevalent at 47.3%, followed by Malay at 30.7%, Indian at 11% and others^1^ (10.9%).

**Table 2:** Risk factors of maternal anemia (*N* = 1,005).

Variable	Overall (*N* = 1,005)
Age at diagnosis (years)	
Advanced maternal age (≥35)	223 (22.3%)
Young maternal age (≤20)	7 (0.7%)
Mean ± SD	31.1 ± 4.67
BMI	
Mean ± SD	27.2 ± 7.08
Median (IQR)	26.4 (7.15)
Obesity (≥30)	251 (26.8%)
History of thalassemia	
Yes	3 (0.3%)
No	994 (98.9%)
Carrier	8 (0.8%)
History of anemia	
Yes	3 (0.3%)
No	1,002 (99.7%)
Ethnicity	
Chinese	473 (47.3%)
Malay	307 (30.7%)
Indian	111 (11%)
Others	109 (10.9%)
Smoking status	
Smoker	26 (5%)

Risk Factors in relation to trimester of anemia diagnosis (*N* = 1,005) *p-*values			
Trimester at diagnosis of maternal anemia	T1	T2	T3
Young maternal age	0.371^†^	1.00^†^	0.364^†^
Advanced maternal age	0.659^†^	0.237^†^	**0.031** ^†^
BMI	0.378^‡^	0.052^‡^	0.480^‡^
Ethnicity	0.991^†^	**<0.001** ^†^	**<0.001** ^†^

† Chi-squared test.

‡ Mann–Whitney U test.

Bolded values are significant.

In univariate analyses (Table 3), advanced maternal age (AMA ≥ 35 years) was significantly associated with third-trimester anemia (*p* = 0.031). However, after adjustment for ethnicity and BMI using Firth bias-reduced logistic regression, this association was no longer significant (aOR 0.94, 95% CI 0.63–1.42). In contrast, AMA was associated with higher odds of anemia in the first trimester (unadjusted OR 1.52, 95% CI 1.10–2.09; aOR 1.40, 95% CI 1.00–1.96). However, this association was not significant in the second or third trimesters after adjusting for ethnicity and BMI.

**Table 3:** Regression analysis of maternal anemia risk factors by trimester.

Risk factor	T1 OR (95% CI)	T1 aOR (95% CI)	T2 OR (95% CI)	T2 aOR (95% CI)	T3 OR (95% CI)	T3 aOR (95% CI)
Advanced maternal age	1.52 (1.10–2.09)	**1.40 (1.00–1.96)**	0.58 (0.34–0.98)	0.63 (0.36–1.09)	0.81 (0.55–1.19)	0.94 (0.63–1.42)
Malay	2.70 (1.11–6.59)	**2.60 (1.10–6.20)**	2.70 (1.11–6.59)	**2.70 (1.12–6.55)**	8.00 (2.12–30.2)	**7.80 (2.10–29.8)**
Indian	3.47 (1.20–10.0)	**3.40 (1.18–9.90)**	3.47 (1.20–10.0)	**3.50 (1.21–10.1)**	14.2 (3.39–59.3)	**14.1 (3.38–59.0)**
Other ethnicity	1.12 (0.18–6.88)	1.10 (0.17–6.85)	1.12 (0.18–6.88)	1.12 (0.18–6.87)	2.60 (0.23–30.0)	2.55 (0.22–29.7)
BMI (per kg/m^2^)	0.97 (0.93–1.01)	0.98 (0.94–1.02)	0.97 (0.93–1.01)	0.97 (0.94–1.02)	0.99 (0.96–1.02)	0.99 (0.96–1.02)

Chinese ethnicity is the reference group. BMI was modeled as a continuous variable (per kg/m^2^). Adjusted models included maternal age (≥35 years), ethnicity, and BMI. Variables with limited subgroup sizes (thalassaemia, smoking, and history of anemia) were excluded from regression analysis due to small numbers precluding reliable estimation. Firth bias-reduced logistic regression was used to mitigate sparse data bias. Bold values indicate statistical significance based on model-derived p-values (*p* < 0.05).

Ethnicity remained a consistent independent predictor of anemia across all trimesters. Indian mothers had the highest adjusted odds of anemia (T1 aOR 3.40, 95% CI 1.18–9.90; T2 aOR 3.50, 95% CI 1.21–10.1; T3 aOR 14.1, 95% CI 3.38–59.0), followed by Malay mothers (T1 aOR 2.60, 95% CI 1.10–6.20; T2 aOR 2.70, 95% CI 1.12–6.55; T3 aOR 7.80, 95% CI 2.10–29.8). Table 4 depicts median Hb levels at T1, T2, and T3, respectively, for each ethnicity group. Chinese women reported the highest levels of median Hb during all trimesters, followed by others (mainly Caucasians), Malay, and lastly Indians.

**Table 4:** Mean (SD); Median levels of Hb (g/dL) among varying ethnicities across the entire pregnancy.

Ethnicity	T1 Hb (g/dL)	T2 Hb (g/dL)	T3 Hb (g/dL)
Chinese	12.3 ± 1.04; 12.5	11.7 ± 1.29; 11.5	12.4 ± 1.42; 12.5
Malay	12.1 ± 1.21; 12.2	10.8 ± 1.15; 10.9	11.2 ± 1.45; 11.6
Indian	11.8 ± 1.27; 12.0	10.4 ± 1.34; 10.7	11.0 ± 1.31; 11.9
Others	12.1 ± 1.34; 12.1	11.1 ± 1.36; 11.4	11.6 ± 1.3; 11.8

BMI demonstrated a mild but non-significant inverse relationship with anemia across trimesters. Variables with sparse data (thalassaemia, smoking, and history of anemia) were excluded from regression analysis due to limited subgroup sizes.

### Maternal and neonatal outcomes

Table 5 summarizes maternal and neonatal complications across the entire pregnancy. Overall complication rates were low in this cohort. Neonatal mortality occurred in 0.4% of pregnancies, and neonatal morbidity in 0.6%, most frequently due to respiratory distress syndrome, low birth weight, or neonatal jaundice. Among maternal complications, antenatal events such as placenta previa and preterm labor were documented in 1.0% of women, while delivery-related complications such as postpartum hemorrhage, cord prolapse, and emergency cesarean delivery occurred in 1.6%.

**Table 5:** Descriptive summary of maternal and neonatal outcomes across the entire pregnancy *n* (%).

Outcome	Overall (*N* = 1,005), *n* (%)
Neonatal mortality (e.g., neonatal stroke)	3 (0.4)
Neonatal morbidity (admitted to NICU for various reasons)	4 (0.6)
Antenatal complications (e.g., PIH/pre-eclampsia, PPROM, DVT/PE, GDM, maternal cardiac arrest)	7 (1.0)
Delivery complications (e.g., PPH, retained product of conception, abruptio placentae, placenta previa)	11 (1.6)

Across the data, there were insufficient cell counts for reliable p-value computation and hence values were excluded.

## Discussion

### Main findings and interpretation

#### Screening and surveillance

The American College of Obstetricians and Gynaecologists recommends that all pregnant women undergo anemia screening in the first trimester, with a subsequent screening between 24 to 28 weeks of pregnancy to facilitate appropriate clinical management (22). The detection of anemia is highest in the first trimester, likely due to early detection at the booking visit with adherence to these screening guidelines, typically through full blood count testing. Physiological hemodilution during pregnancy may also explain the highest rates of maternal anemia in T1 and T2. Although red blood cell (RBC) production increases during pregnancy, plasma volume increases at a faster rate, leading to decreased Hb and haematocrit values (22–24). To support normal fetal growth and development and anticipate blood loss at birth (25), maternal blood volume increases by approximately 40–50% during singleton pregnancies, while RBC mass increases by only 15–20% (22).

Despite most cases of anemia in pregnancy being physiological, it remains crucial to differentiate it from other treatable causes, such as iron deficiency anemia (IDA), aplastic anemia, autoimmune haemolytic anemia, and inherited conditions like sickle cell disease and thalassemia.

Nutritional factors may also play an important role, as iron requirements increase substantially during pregnancy and may exceed dietary intake, particularly in the absence of adequate supplementation. However, as iron studies and dietary data were not routinely available in this retrospective cohort, we were unable to directly assess the contribution of nutritional deficiencies. Future prospective studies incorporating iron indices and dietary assessments would better elucidate these mechanisms.

Our study found the prevalence of maternal anemia to be the highest in the first two trimesters. The third trimester prevalence was 23.4%, a significant value, though only with a corresponding 7.9% detection rate. This underscores the importance of continuous evaluation throughout trimesters, especially for high-risk individuals, such as those with a history of maternal anemia in previous pregnancies. Therefore, we advocate for more frequent screening and enhanced patient education regarding the signs, symptoms, and consequences of anemia.

#### Comparison with regional studies

Our prevalence of maternal anemia (66.2% during pregnancy, with 36.9% in T1, 37.0% in T2, and 23.4% in T3) is considerably higher than reports from Thailand. In a 2023 prospective Thai study, rates of anemia were much lower—6.92% at first antenatal visit, 24.6% in the third trimester, and 4.76% intrapartum (26). These differences may reflect population demographics, dietary patterns, antenatal surveillance practices, and variation in anemia definitions. The absence of routine iron studies in this cohort limited direct aetiological comparisons.

#### Management: addressing risk factors

Key risk factors identified in our cohort include AMA in the third trimester and ethnicity in the second and third trimesters.

### AMA

In our dataset, AMA was associated with anemia only in the first trimester, suggesting that age-related physiological or nutritional factors may exert greater influence early in gestation but become less pronounced as pregnancy progresses. This may reflect improved nutritional supplementation and clinical surveillance later in pregnancy.

AMA has long been studied to be associated with less favorable obstetric outcomes including a higher risk of maternal anemia, potentially due to factors such as increasing parity, poorer nutritional status, and overall health (21). Managing these patients in high-risk obstetric clinics could be beneficial.

### Ethnicity

Ethnicity was the strongest and most consistent independent determinant of maternal anemia across pregnancy. Both Malay and Indian mothers demonstrated significantly higher adjusted odds of anemia compared to Chinese mothers in all three trimesters. This finding aligns with regional data suggesting persistent ethnic disparities in hemoglobin levels, likely influenced by a combination of genetic, dietary, and socioeconomic factors.

From existing literature, certain ethnicities are predisposed to higher rates of maternal anemia. Ethnic minority background has been identified as an important risk factor for IDA (27). In Western societies, ethnicities at risk included Hispanic people and Black people (28). Our study, focused on an Asian population of Chinese, Malays, and Indians, found that Indian women had the highest rates of anemia, with the lowest median and mean Hb levels. These findings are consistent with other studies (13, 29, 30). More specifically, studies which took IDA into account revealed that the Indian race was associated with a significant lower level of serum ferritin (11).

In developing countries, factors like low education levels, socioeconomic status, and vegetarian diets increase susceptibility to maternal anemia (31). While these factors are less likely to be primary causes in developed countries like Singapore, further studies are needed for Malay and Indian women. Potential contributing factors include dietary deficiencies in iron or Vitamin B12 attributed to cultural or habitual reasons, particularly among vegetarians (32–34); or lower socio-economic status as it correlates with lower health literacy levels as well (35, 36) which might cause late antenatal booking and reduced detection rates from routine testing (37). Reproductive health factors such as multiparity and shorter inter-pregnancy intervals were also significant, with Indian women being most likely to be multiparous (followed by Malays and lastly Chinese) in a local study (38).

The discrepancy in terms of maternal anemia rates reveal the need for re-evaluation in terms of management strategies for local Indian and Malay women. We propose increased screening and nutritional strategies, including iron supplementation and dietary guidance, in collaboration with dietitians. Enhancing health literacy through patient education on anemia in pregnancy is also essential.

### BMI

Previous research has shown that higher early pregnancy BMI is associated with higher Hb levels, reducing the risk of anemia (39), while underweight women are more susceptible to severe anemia during gestation age of ≤20 weeks (31). However, in our cohort, we did not establish any association between abnormal BMI (<18.5 or >25 kg/m^2^) and maternal anemia incidence. There has also been a paucity in research regarding the direct relation of BMI to maternal anemia incidence in varying groups of women. Maintaining a healthy BMI in terms of Asian standard 18.5-25 kg/m^2^ should be recommended to mothers throughout pregnancy as a representation of adequate nutrition and caloric intake.

#### Neonatal and maternal outcomes

In our cohort, overall complication rates were low across the study population. Our outcomes were analyzed descriptively due to low event rates, incomplete longitudinal hemoglobin data, and the absence of a non-anemic comparator group. Given the rarity of events, formal statistical comparisons were not performed and causality was not inferred.

Existing literature has demonstrated associations between maternal anemia and adverse maternal and neonatal outcomes, including preterm birth, low birth weight, and perinatal mortality. Large population-based and meta-analytic studies in South and Southeast Asian settings have reported increased risks of these outcomes among anemic mothers compared to non-anemic mothers (40, 41).

Nonetheless, these limitations highlight the need for prospective studies with complete longitudinal data and adequate statistical power to better define these relationships. They also reinforce the importance of early antenatal screening and appropriate management of maternal anemia across the entire pregnancy to minimize maternal and neonatal morbidity.

#### Strengths and limitations

The exclusion of thalassaemia carriers, smokers, and women with a prior history of anemia was necessitated by low subgroup numbers, which limited statistical reliability. As pregnancies conceived via assisted reproductive technologies were excluded, our findings may not be generalizable to this population as well. Larger-scale studies are required to assess these potential contributors more robustly.

Our retrospective design enabled the evaluation of a large consecutive cohort, providing a comprehensive overview of maternal anemia patterns in our setting. However, this design inherently limits causal inference and may be affected by missing data or inconsistent documentation. A key limitation was the inability to perform a detailed subanalysis of anemia subtypes, as iron studies (ferritin, serum iron, transferrin, total iron binding capacity, and iron saturation) were not routinely performed in our institution. Despite the sizeable cohort, this limited our ability to distinguish iron deficiency anemia from other pathological causes. In addition, all thalassaemic mothers were excluded from the analysis, which may underestimate the true burden of anemia in pregnancy. Future prospective studies incorporating comprehensive hematological indices would better characterize the etiologies of maternal anemia in our local population.

## Conclusion

Maternal anemia remains highly prevalent in this multi-ethnic Asian obstetric cohort, particularly in early pregnancy and among Indian and Malay women. Advanced maternal age was associated with first-trimester anemia.

These findings highlight the importance of early and sustained antenatal screening, risk-aware surveillance, and targeted nutritional counseling to reduce preventable maternal and neonatal morbidity. Future prospective studies incorporating iron indices may further refine risk stratification and inform personalized antenatal care strategies.

## Data Availability

The original contributions presented in the study are included in the article/supplementary material, further inquiries can be directed to the corresponding author.

## Footnotes

1 Others included Caucasians, Thais, Vietnamese, and Filipinos.
